# The Perception of Biological Experience in Patients With Major Thalassemia: A Qualitative Research

**DOI:** 10.5539/gjhs.v7n1p79

**Published:** 2014-08-15

**Authors:** Azizollah Arbabisarjou, Tahmineh Karimzaei, Abdolghaffar Jamalzaei

**Affiliations:** 1 Pregnancy Health Research Center, Zahedan University of Medical Sciences, Zahedan, Iran; 2 School of Health, Iranshahr University of Medical Sciences, Iranshahr, Iran; 3 Emam Ali Hospital of Chahbahar, Zahedan University of Medical Sciences, Zahedan, Iran

**Keywords:** perception of biological experience, patients with thalassemia major, qualitative study

## Abstract

**Background:**

Thalassemia Major Disease is not only assumed as a health disorder, but also a social- economic problem in many countries. The costs of transport and preparation of drugs is considered as the greatest problems for more than 63.8% of the patients’ families.

This study was conducted by aiming at describing biological experience among parents of patients with Thalassemia Major.

**Method:**

The current qualitative investigation was carried out on 32 parents of patients with Thalassemia Major and by means of unstructured interview in- depth through snowball sampling technique in 2013. The data were analyzed by conventional content analytical method.

**Findings:**

The perception of biological experience of parents of patients with Thalassemia Major were classified based on participants’ experiences into three main themes including psychological experiences, physical experiences, and social experiences. 1) Psychological (mental) experiences comprise of two subclasses of the reduced self-confidence, deficient emotions and negative emotions; 2) Physical experiences consist of three subclasses of sleeping disorders, pains in various parts of body, and limited physical activity; and 3) Social experiences includes 3 subcategories of interpersonal relations, reduced income, job, and limitation in doing tasks. 4) Treatment experiences comprise of five sub-themes including 1- Shortage of drugs, blood, and filter etc; 2- Less experienced personnel; 3- Lack of training the patients’ parents by personnel in thalassemia ward; 4- Lack of visiting patients by physician in thalassemia ward; and 5- Inappropriate behavior of personnel toward patients and their parents.

**Conclusion:**

Thalassemia Major has affected negatively on several fields of health for these patients and their parents including physical, mental, economic, and social areas. Reducing these problems requires constant interventions and surveying health and medical status of these patients.

## 1. Introduction

Thalassemia is the most epidemic type of hereditary chronic anemia among humans, which is created due to imbalanced production of peptide cycles as constituents of hemoglobin (Haghshenas & Zamani, 2000; Karimi, Ghavanini, & Kadivar, 2000). Thalassemia is divided into two groups based on type of reduced hemoglobin cycle: Alpha and Beta (Tarbiat Modares University, 2004) and it is transferred by autosomal recessive allele so that if two persons, as carriers of thalassemia, get married together there is 25% probability for their children to suffer from thalassemia major while 25% of their children will be healthy and the rest 50% are carriers for thalassemia. Various types of thalassemia may be seen almost in all points of the world but this disease is more prevalent at the seashores of Mediterranean Sea, equator, African and Asian continents remarkably in such a way that the region which ranged from beaches of Mediterranean Sea (Italy and Greece), throughout Saudi Arabia, Turkey, Iran, India through southeastern Asia including Thailand, Cambodia, southern China, and Burma are called Thalassemia Belt. The genetic incidence is 2.5-15% in these areas. Among them, alpha thalassemia is mainly observed in southeastern Asia as well as western African beaches and also its incidence has been reported 4.8-10% in Thailand. In this course, more than 50% of total population suffers from a type of alpha thalassemia in desert areas in eastern Saudi Arabia. Alternately, about 3% of total world population is carriers for beta thalassemia where these gene-carriers are further seen in Italy and Greece but there are also these carriers in northern and western Africa, Iran, Saudi Arabia, Pakistan, and India (Haghshenas & Zamani, 1993; Karimi, Ghavanini, & Kadivar, 2000).

In Iran, thalassemia, particularly type Beta, is more prevalent and its incidence is more noticeable especially in northern areas, Fars, Baluchestan, and some other zones (Ministry of Health and Medical Education of Iran (1997-2001).

In other words, approximately 25’000 people suffer from thalassemia major in Iran (Ghotbi & Tsukatani, 2005). The frequency of patients with thalassemia varies from 3 to 100 people per 100’000 in several provinces. Sistan and Baluchestan province in southeast of Iran, with 2’700’000 population includes 2300 patients with thalassemia major (85 per 100’000 people). The frequency of minor thalassemia is reported to be 10% in northern areas and 4-8% in other places. The number of patients suffering from thalassemia in Sistan and Baluchistan Province in southeast of Iran have been estimated 1167 in 2001 (Naderi et al., 2012; Alavi et al., 2007). At present, Sistan and Baluchistan with 2050 major thallassemic patients in a subtropical area in the southeast of Iran holds the highest rate of thalassemia in the Iran (Mirimoghaddam et al., 2012). These patients may meet their annual medical needs by transfusion of 50’600 blood units. About 72’000 blood units are donated in Sistan and Baluchestan province every year among them 70% of these donated blood units are used for thalassemic patients while approximately 25% of the donated blood is consumed for thalassemic patients throughout the country (Abolghasemi et al., 2007). Although blood transfusion may increase life period in beta thalassemic patients, there is no physiologic mechanism to chelate iron. Chelation therapy is vital to prevent major organ damages and it must be monitored to prevent exacerbating existing renal dysfunction due to thalassemia disease itself (Naderi et al., 2013). Following to several blood transfusions, accumulation of iron in various tissues and limbs may lead to fibrosis and it causes cells death and inactivation of the given organ. Among the involved major body organs, heart, liver and kidney (Torcharus & Pankaew, 2007), kidney and secretory glands are maximally damaged so that if patient tolerates anemia at least s/he will be died due to cardiac and glandular deficiency (Zendehbad et al., 2009; Rund & Rachmilewitz, 2005). According to the investigation that has been conducted by Shahla et al. (2003), of 1069 patients with thalassemia major (Beta) 16 patients (1.5%) were died due to severe cardiac traumas and 19 patients with severe cardiac disorders went under treatment by electrocardiogram and monitoring system while 106 patients (9.9%) suffer from cardiac disorders at average level. Thalassemia is not only a health disorder but it is an economic-social problem in many countries (Mirimoghaddam et al., 2004). The costs of transport and preparation of drugs were the highest economic problem for more than 63.8% of families of these patients (Merat et al., 1993).

In a study, which has been carried out by Rafizadeh titled “The exploration into the rate of awareness of pre-university and high school students from Gilan Province of thalassemia”, they selected a statistical population among male and female high school and pre-university students within a descriptive survey by means of randomized sampling technique and from various cities in Gilan province at academic year 2005-6 during execution of this study, the findings indicated that the rate of awareness about thalassemia disorder among male and female high school and pre-university students has been at low level (11.07-20%) and this is fact given that Gilan province is enriched in terms of abundance of thalassemic genes (Rafizadeh et al., 2010).

In the investigation that was done by Jafari and his colleagues (2006) under title of “The prevention from thalassemia by rising level of awareness among spouses and avoidance of carriers from getting married in Gorgan city”, this cross- sectional study was conducted on samples to measure the level awareness among 282 participants from pending married spouses. Similarly, 107 thalassemic minor spouses were identified in terms of avoidance from the marriage during period 1997-2003 but despite of their awareness at good level, the performance of the given spouses was not appropriate and a greater percentage of thalassemic carriers took the more risky marriages.

In a survey that was conducted by Razieh (2012) titled “The investigation in the effect of training based on healthcare doctrine model in prophylactic behaviors against thalassemia major among thalassemic carrier spouses”, in this semi-empirical study, 50 thalassemic carrier spouses were elected by classified random sampling method among thalassemic carrier spouses under the supervision of Hirmand medical- healthcare centers as the case group and 50 thalassemic carrier spouses were chosen from Zahak city as control group. The findings showed that training based on this model might increase prophylactic behaviors against thalassemia in the case group but the control group, which were only trained for the general courses showed no change in behavior of spouses. Thus, employing this model may be effective in the current training courses of healthcare system.

In study of Torcharus and Pankaew (2011) titled “The review of quality of thalassemic children’s life”, which has been carried out on 49 thalassemic patients under treatment by iron-chelator drugs, the results indicated that a significant difference was observed among the intensity of disease symptoms, age of starting these symptoms, and hematocrit level in patients with quality of their life.

In a survey that was done by Wahabi and colleagues (2010) entitled “The study on attitude of parents and family members of patients with thalassemia major”, as a descriptive study on parents of patients at age 8-22, who had referred to hospital of thalassemia in Malaysia, the results showed that the given disease may cause worry and adverse effect on educational status and underachievement in students.

Therefore, given that the qualitative research is conducted to describe and increase perception of human’s experience and it is assumed as a method to acquire awareness and insight by exploration of concepts while this insight is not acquires by finding cause-and-effect relationship but it is achieved due to enhancing individual’s perception from the given totality so this type of investigation possesses exploratory nature and it is intrinsically explanatory in which the numbers are used to describe a phenomenon instead of letters (LoBiondo-Wood & Haber, 2006) as a result examining human’s spirits by numerical and quantitative values is a difficult task. Thus, it seems that to review the spiritual reactions and responses based on the few qualitative studies about this subject in Iran; the current research was carried out in order to recognize live and tangible experiences from parents of patients with thalassemia major.

## 2. Methods and Materials

The present qualitative research was conducted with phenomenological approach and conventional content analysis method. In this technique, the information is acquired directly from the studied participants without imposing the predetermined classes and or previous theoretical attitudes. The created knowledge by this method is based on the unified and unique attitudes from participants and derived from textual real data. In other words, in this technique codes and classes are extracted directly and in deductive from raw data (Hsieh & Shannon, 2005).

The participants were chosen from parents of patients with thalassemic major, who were Iranian Muslims with Persian dialect, based on snowball sampling according to the determined criteria.

The criteria for entering in this study comprised of having child with thalassemia major, alertness and tendency to express their own internal emotions about research subject, being at age 15-49, ability to speak Persian, not suffering from the verified psychological diseases, cognitive disorders, blindness and deafness, and access to telephone system. With respect to domain of experiences and process, selection of samples was continued until no new data was acquired during trend of acquisition of data. In this study, data were saturated and completed with 30 participants. But in order to make sure further, two other participants also participated in this interview. Then, the other operations were done including rewriting, encoding, and extraction of minor and major classes.

The unstructured and in- depth interview was the main method for data collection. The interview was started with a wide and general question about their daily life experiences and then some exploratory questions were asked to encourage participant and achieving deeper information. The period of interviews varied among 45-60min with number of 2-5 sessions. The participants were asked to personally determine time and location of interview arbitrarily. The interviews were continued until acquisition of deep data. With taking written permission from participants, the conducted interviews were recorded on reel (video) and immediately after the end of interview; the recorded tapes were analyzed and rendered after watching and listening to interviews for several times. To separate thematic sentences in this investigation, the holistic and selective approach was taken. As a result, the body of an interview was noticed as a whole from the beginning of it and the fundamental concept or major purpose of this text was described as a totality or within a few clauses (holistic approach). Study on interviews holistically led to create 32 descriptions from total interviews with 32 participants. Afterwards, text of any interview was read for several times and those sentences or phrases, which seemed to be relevant to the described phenomenon or revealed it, were selected (selective approach). Separation of thematic sentences and their conversion and changing of the given sentences were done individually for any interview (empirical or primary theme). The primary classes of design and themes were embedded in those classes and by change and replacement of themes, merging common and overlapping themes and omission of irrelevant themes, the common basic themes appeared. In other words, phrases, sentences, and clauses belonged to each of interviews were organized separately from other interviews and with respect to commonalities and within the framework of themes and sub-themes. Using the experts’ comments for study and verification of themes also guided the researchers in achieving depth of the purposed concept by the participants at several steps. These themes were reduced gradually with deletion of similar and overlapping themes at next steps and finally 9 sub-themes and 3 themes as main themes resulted.

To continue this trend, in order to confirm the validity and accuracy of study, reliability, confidence, and their potential for validation were examined. To guarantee the reliability of this survey, findings of this study were presented to participants and they expressed their opinions to researchers about the coordination of findings with their own experiences. Likewise, the participative thinking and pondering was done concerning to the appeared themes by research team during several stages of investigation. The researchers guaranteed the validation of this study by keeping documentations during all phases of this survey. Some of the other guarantee factors for validation of this study were researchers’ interest in the studied phenomenon, long term connection with data and also trying to acquire others’ opinions in this regard. Moreover, the present study has been conducted by teamwork and by advice and supervision of some experts and this may both provide reliability of data and validation of them.

In order to observe moral consideration and before starting interview, the participants were informed about research goals and importance and they participated consciously with consent in this investigation. They were asked to permit for participation in this study and using video camera to record their interviews and also they were made assured the acquired information would be only employed for research goals and it would not be put at disposal of other party rather than researching team. Similarly, it was confirmed this point to the participants that they could leave the process of research and participation in this study whenever they announced and privacy of their characteristics reserved confidentially during research and after the investigation.

## 3. Findings

Of the conducted data analysis in this study three main themes have been derived from participants’ attitude, thereby they could draw the perception of experience in parents of thalassemic patients. These themes included psychological (mental) experiences, physical experiences, social experiences, and treatment experiences. 13 sub-themes were totally extracted from these themes so that the psychological themes comprised of 1) reduced self-confidence; 2) reduced recess and amusement; and 3) negative feelings and emotions. The physical experiences included three sub-themes: 1) sleeping disorders; 2) pains in several parts of body; and 3) restriction in physical activity. Social experiences consisted of two sub-themes: 1) reduced income and job (housing and transport higher costs), and 2) interpersonal relationships. Treatment (therapeutic) themes included five sub-themes: 1) shortage of drugs, blood, and filter etc; 2) less-experienced personnel; 3) lack of training patients’ parents by personnel in thalassemia ward; 4) non visiting o patients by the physician in thalassemia ward; and 5) personnel’s inappropriate behavior with patients and their parents.

### 3.1 First Theme: Psychological Experiences

This theme consisted of three sub-themes under titles of reduced self-confidence, reduced recess and amusement, and negative emotions.

#### 3.1.1 The Reduced Self-Confidence

The participants with this disorder had experienced deficiency in self-reliance, lack of trust on their own, attaching less value for their own, self-dissatisfaction, and disappointment toward the future. Generally, some of participants expressed their opinion in this regard:

“My husband told me: Whatever you do you cannot treat this child thus quit it so I abandoned this trend.” (Participant no 6)

“Since the time when they connected angiocath to my child, if you sit here and listen to his words at night you will be frustrated like me. If you heard the great question marks, which my child encountered you would be unhappy as a human for him.” (Participant no 11)

“This is my child with major type, who is more cute and lovely for me than my other children. When he tells me his body is full of pains that time I become mad. I wished God did not give me such a child. I wished God gave me a healthy one so if He gave me a child He did only healthy or nothing better.” (Participant no 8)

“If someone is fertile is better than to suffer from thalassemia major. We have no intelligence, no head, and no nerves. By god! We became mad for thinking about the problems of these children. I could not to do anything.” (Participant no 14)

#### 3.1.2 Reduced Recess and Amusement

Most of participants had experienced fatigue and tiredness in recreational activities. Generally, a participant expressed in this sense: “After finding disease in my child, I had no time for amusement and recess because I had to bring the child to physician and for blood transfusion or from this town to that city in order to find his drugs so that does it remain anytime for recess?!” (Participant no 32)

(Mother of two patent children is sobbing and crying) “When these children catch headache half of body of their mother is melt. Now I am nervous so I take pill of nervousness. My head and body burn and all my bones burn as well from sorrow for these children. I hope God not to give anyone this sadness. So I would not like for amusement.” (Participant no 27)

#### 3.1.3 Negative Feelings and Emotions

The participants often tended to recall the diaries of sense of guilt and repent that had been experienced. For example, some of participants implied in this way, “I extremely felt sense of guilt for which I did not pass PND medical test. When I was in Zahedan those days (before my husband who was hospitalized for accident there) I was in a dream that my child was healthy. This is only my daughter, I worried for her a lot … (Mother was crying so disrupted her words. “ (Participant no 29)

“I was extremely blamed in the family for this point that all of the other members who had not been already tested before marriage had healthy children but I observed this advice while my child became thalassemic major so this caused me to be criticized. (The mother stares at her front in this time)” (Participant no 11)

### 3.2 Second Theme: Physical Experiences

This theme included three sub-themes: 1) sleeping disorders, 2) pains in various body organs, and 3) restricted physical activity.

#### 3.2.1 Sleeping Disorders

The participants have often experienced insomnia and lesser sleeping in their children. For example, some participants have expressed in this regard: “When they transfuse blood to our bodies they are annoyed up to morning so we are awakening along with the children.” (Participant no 18)

#### 3.2.2 Pains in Various Body Organs

Many participants have experienced several pains in various organs of their children’s bodies such as headache, backache, abdominal pain, and foot pain etc. For instance, some participants express in this way: “From there (i.e. thalassemic ward after blood transfusion) the patients catch fever and headache and afterward they lie down in the home for one and half a day neither eat anything nor stand up at all.” (Participant no 27)

“The child mainly suffers from backache, become bruised, and with headache.” (Participant no 28)

#### 3.2.3 Restricted Physical Activity

Most of participants have experienced the sense of fatigue, weakness, and disability in their children during daily activities such as activity in school, physical activity, and religious performances. For example, in this sense some participants implied that:

“My child likes to work and to earn for his life but he gets tired soon and no suitable work for him proportional to his status.” (Participant no 15)

### 3.3 Third Theme: Social Experiences

This theme comprised of two sub-themes including the reduced income and job and interpersonal relations.

To describe their own experiences, the participants expressed some factors like lack of suitable job and inadequate wages and lower financial solvency to meet patient’s needs. For example, concerning to this issue, some of participants implied: “My husband is a taxi driver and he has another wife. He does not bring this child for his drugs and he says I have no money to take my child for treatment to Zahedan.” (Participant no 7)

“Now we should bring this child for blood transfusion to a hospital outside the city but taxi fare is expensive. How could a worker live with five children this time?” (Participant no 10)

“The medical test fee for this child is 1’000’000 Rials. Could anyone go to Zahedan who has not any money to pay for taxi fare in order bring his/her child from the village (to come Iranshahr) for blood transfusion in order to do the medical examinations every three months to this city? “ (Participant no 9)

#### 3.3.1 Interpersonal Relations

Participants expressed their experiences in their relations with their spouses, child, and patient with thalassemia major with family members. For instance about this subject, some participants expressed: “If the relationship is highly good among wife and husband and they were lovers before marriage after bearing a child with thalassemia major this relationship will be disrupted.” (Participant no 21)

“My child is very nervous and he continually quarrels with his brothers.” (Participant no 13)

### 3.4 Fourth Theme: Participants in Description of Their Experiences

#### 3.4.1 Treatment (Medical Experiences)

It included five sub- theses under titles of 1) shortage of drugs, blood, and filter etc; 2) less-experienced personnel, 3) lack of training parents of patients by personnel in thalassemia ward, 4) non visiting of patients by physician in thalassemia ward, and 5) personnel’s inappropriate behavior with patients and their patients.

#### 3.4.2 Shortage of Drugs, Blood, and Filter

The participants described their experiences about shortage of drugs and blood etc. For example, about this matter most of participants expressed: “It is very extremely tensely that when we refer to hospital they told us they have not sufficient blood to transfuse for the child.” (Participant no 13)

“All drugs, which have been prescribed by physician, we bring its prescription to center of medical counseling in order to be sealed but when we bring it to drugstore, they tell us we have not these drugs so children have not taken these drugs for 1-2 months.” (Participant no 21)

“They transfuse blood to children without filter so that they lie down at night without eating anything with headache and hand and foot pains after transfusion but if they transfuse blood with filter the children have better conditions. They connect one end of a hose like a serum.” (Participant no27)

“My daughter has suffered from cardiac disorder during this period and she has to inject five vials every night but he has not injected these vials for five months because whenever we refer to drugstore they told us that they ran out of drugs now or said us to refer to them next Wednesday so when we go there two or three days later they replied us the last prescription was terminated.” (Participant no 25)

#### 3.4.3 Less-Experienced Personnel

Many participants have been exposed to the less- experienced personnel. For instance, some of them implied in this regard: “The personnel, who have been employed there, are beginners so they do not know problems of children and they ignore the patients if their bodies are swollen or irritated and or they have headache so they say we do not know. (Participant no 17)

#### 3.4.4 Lack of Training Parents of Patients by Personnel in Thalassemia Ward

Most of participants had experienced lack of training for parents of patients by ward personnel. For example, some of participants implied: “Here nobody cares for patient at all they do not train patient well and they only tell us that your children should not cry and spoil. But they tell us only this words and nothing to our child about disease.” (Participant no 17)

#### 3.4.5 Non-Visiting of Patients by Physician in Thalassemia Ward

## 4. Discussion

The patients with thalassemia major are the humans, who should be under medical care throughout their lifetime. It is crucially necessary and important to assist them by some of relatives like parents during treatment trend and controlling disease. This study indicates that parents’ life is extremely influenced by this disease in terms of psychological, sociological, and economical aspects and research findings signify the reduced self-confidence, sense of guilt, feeling of disappointment, and painful and sad emotions in parents, which are intensified by observation of visible physical changes in their children and they are followed by some consequences regarding creation of social interactions and ties and disease in their children may affect on quality of parents’ life, who are responsible for their care.

In a study (Prasomsuk et al., 2007) that was carried out by means of qualitative method to conduct in-depth interview concerning to 15 mothers with thalassemic children. The findings showed that all mothers believed that disease in their children has importantly affected on their life. Six cases of the subjects, which appeared during review of findings, included shortage of information about thalassemia, social- psychological problems the mother had experienced, those financial problems which the mother were involved in, their concern about future, social supporting systems, and efficiency of systems for presentation of medical cares. These mothers suffered extremely from social stress because the quality of their children’s life had been negatively affected by the disease; for example, due to the absence of their children from school or the problems caused by tolerating the pains during treatment processes, their physical constraints and restricted activities. The findings confirmed and recalled the necessity for making some efforts like empowerment of mothers to tackle with social stresses in living with thalassemia.

Also, this investigation revealed some points about the relationship with disease effect on formation of social- mental problems such as concern about body image of children, worry about the disease itself and also concern about future of children among these mothers. The impact of Thai culture about people’s ideological values and beliefs, which are expressed concern and respect regarding others, was also evident in the mind of these mothers. Although, according to altruist moral principles, they might declare they tried to inform and warn other mothers about prevention from occurrence of such problems.

In advanced countries, the patients and their relative, who are responsible to take care of them or related to them, are not left alone. Usually, several support groups are formed in the society and local society or in neighbor area in order to give potential to these individuals to fight against disease and its problems and difficulties. The interaction and dialogue with others, who are familiar with or involved in these difficulties, may help the patients to learn new strategies and to become more capable in this respect (Veiel & Baumann, 1992; Haddow, 2005; Dyson, 2007).

In findings about psychological experiences, some factors such as reduced self-confidence, deficiency in recess and amusement and (negative) feelings and emotions were found among 95% of participants. Concerning to physical experiences, factors of sleeping problems, pains in several body organs and limited physical activities were evident in 94% of participants. In social experiences, variables of reduced income and job, cost-consuming nature of medical care for their children and the subsidiary costs might exert pressure on their parents and also the interpersonal relations of parents are effected and it caused the sense of guilt in parents and dispute among them.

Ghanizadeh et al. (2006) in psychological ward at Hafez hospital in Shiraz city in their report titled as the prevalence of psychological disorders, depression, and suicidal behavior among children and teenagers with thalassemia major have referred to this point that there were some psychiatrist symptoms at common level and no higher level of suicide- related behavior has been observed than what it applies to all people in the society.

The study that was carried out by Ekhtiari Amiri (2006) about patients with special diseases (thalassemia, homophily, and diabetes etc) in Fereydun Kenar Hospital of Imam Khomeini for evaluation of family’s performance and correlation and mental status of special patients indicated that performance of family affected on their mental state. The family may be assumed as the paramount effective social institution on behavior and personality. The performance of family may be followed with many impacts in incidence of mental disorders in persons or their mental health.

This investigation showed that in medical experiences, some problems such as shortage of drugs and blood filter as well as problems in their preparation and shortage of some blood groups and irregular distribution system have caused wasting time and their satisfaction regarding medical system and inadequate training of parents and patients by physicians and personnel and also lack of daily visiting of them by physician caused complaints among parents. This study and other researches, which show the necessity for rising awareness, development of trainings about reality of disease and providing and supply social supports throughout the society to fight against psychological consequences and stresses caused by involvement in disease among the given patients and their relatives and familiars may affect on duration of concerns and stresses about experience of disease and the processes of treatment for family members at present and in the future and also influence in therapy trend and improvement of their physical and mental health.

Making effort to provide social support, which may positively affect on life process of parents under disease conditions, requires covering not only a limited area, but also taking some measures in formal training course as well as employing mass media regarding this disease and as some part of supportive efforts, it necessitates making some social efforts to reduce financial pressures in which the family is involved.

## 5. Conclusion

The of this study may be employed to improve design of training programs by providers of medical- healthcare services as well as in the course of codified and executive planning to examine, recognize, and prevent from bearing child with thalassemia Major. With respect to growing trend of thalassemia major in the country, it necessitates taking appropriate strategies to remove improper perceptions among the public in society, family members, and treatment team and to be designed and executed thereby the problems of thalassemic patients to be at least minimized. Hence, the requisite for more exploration in improper perceptions and their correction by public training through mass media may be felt as well. With respect to the aforesaid issues, these plans should be integrated with training programs and interventions in change of behavior. The derived information from this study may be utilized as the subject for other investigations in this regard in order to open other horizons and perspectives concerning this disease.

### 5.1 Constraints

The foremost restriction in this research was spiritual and mental conditions of participants in interview, which might affect on trend of interview and responses. In this sense, it was tried to control this constraint by creation of friendly climate and through attraction of their trust to some extent.
